# Radical Knowledge Management: Using Lessons Learned From Artists to Create Sustainable Workplaces

**DOI:** 10.3389/frai.2021.598807

**Published:** 2021-07-13

**Authors:** Stephanie Barnes

**Affiliations:** Independent Researcher, Berlin, Germany

**Keywords:** knowledge management, VUCA, arts-based initiative, sustainable workplace, creativity, innovation, sustainability, organisational change

## Abstract

This study weaves together research that has been published over the last 20 years and creates a narrative about how we can change our organisations so that they are fit-for-purpose in the 21st century. Using knowledge management as the starting point, the question “How do we move forward in a sustainable, holistic way to create organisations that are healthy and balanced among social, environmental, and financial performance (triple bottom line)?” needs to be answered. This brand new form of knowledge management is called radical knowledge management (radical KM).

## Introduction

The objective of this study is to review the existing literature^1^ and combine it with the author’s anecdotal observations to make recommendations in support of changing people’s behaviours and attitudes so that our organisations transform to be successful in the 21st century. It also aims to push knowledge management practitioners towards taking a more active role in the facilitation of knowledge creation and not just protectors of the past and the status quo.

The analysis starts from the premise that in the new work^2^ we are all leaders and that, to be successful, we must use the knowledge and tools available to us in creative, innovative ways, connecting the dots in ways not previously imagined. To do this, we must use critical thinking, reflection, and resilience in a sustainable way to continually adapt to the volatility, uncertainty, complexity, and ambiguity (VUCA) in our environment—these practices are the underpinnings of knowledge management.

The new work requires us to be sustainable. It is concerned with recognising that the way we have been working is not working, we are not machines, we are not production lines, and we are not automatons. The new work requires the whole person to be involved in their work, not just part; it requires on-going learning and engagement; and it requires creativity and self-fulfilment. Many of these things are learned through adopting a creative and artistic approach, as discussed in this article.

**FIGURE 1:**
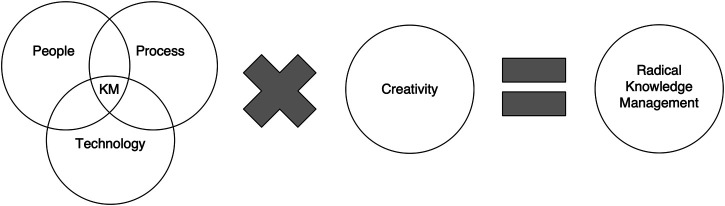
Radical knowledge management.

This study provides a hypothesis as to how we can better enable this in each other and our organisations. It also ties together some apparently disparate ideas: new work, innovation, creativity, resilience, sustainability, and knowledge management to create radical knowledge management (radical KM) (Figure 1). What is the common thread that allows this to happen? People, that is obvious, but additionally, they all have to do with changing/adopting behaviour, focusing less on the part and more on the whole. While it is possible to change people’s behaviours using checklists, books, advertising campaigns, and any number of other targeted, specific activities, what if there was a way to help people develop/evolve these behaviours that was more enjoyable and experiential and helped bring about a more balanced, sustainable approach in the process?

Ultimately, this is a concept article. It is a call to action for knowledge management to step outside the box and consider the needs of knowledge workers. What do knowledge workers need in order to be successful in the 21st century? What will help them deal with the volatility, uncertainty, complexity, and ambiguity (VUCA) of our organisations and economic systems?

While a balanced approach to people, process, and technology is what makes knowledge management successful, technology is, at best, an enabler, not the end point or even the point of knowledge management. Knowledge management is about connecting people to the knowledge they need to do their jobs. In the past, this has been about processes to extract and share knowledge and the technology to store and facilitate knowledge transfer. It has been about stores of knowledge; however, the further the society moves into the knowledge and digital age, the less it needs stores of knowledge. Stores of knowledge maximized efficiency and effectiveness but minimized creativity and innovation. Artificial intelligence (AI) drives this even further by being able to learn and perform human-like tasks.

One of the things that differentiates humans from AI is our creativity (Porter and Heppelmann, 2019), and yet, this is something that has been educated out of us in our focus to be efficient and effective. It is knowledge management’s job to bring it back, to facilitate it within our organisations, and this is the knowledge that people need in order to do their jobs in the 21st century. In the age of AI, people will do the things that AI is not good at: creativity (Wilson and Dougherty, 2019).

As a first step, let us consider sustainability and why it is one of the missing pieces in many of our organisations and why it is part of this radical new approach to knowledge management.

## Sustainability: What Is It, Why Is It Important?

Through the adoption of Taylorism [“Taylor focused the ideas of Whiney and Ford used for parts and chassis and applied them to the workers themselves.” (Austin and Devlin, 2003)], many of our organisations have become increasingly unhealthy, treating the people who work for them as automatons, reducing and eliminating any room for reflection, experimentation, and being a whole person (Austin and Devlin, 2003).

While the production-line approach to work may have been satisfactory in the 20th century, where knowledge was more stable, the early years of the 21st century have revealed a near-constant state of change and flux (as the on-going COVID-19 pandemic keenly demonstrates). This requires a different approach, one that engages the whole person, not just part. It requires an approach that gives autonomy to the doers/makers in order to adapt and change as the environment changes. It requires constant learning and a focus on the tacit rather than the explicit (Thomas and Seely Brown, 2011). This is the way forward for our organisations.

In reviewing the literature on art and sustainability, most, if not all, of it appears to be about using art projects to draw attention to the issues of sustainability. For example, using plastic wastes to make sculptures and other pieces of art to draw attention to the plastic that fills oceans, landfills, and landscapes (Jónsdóttir, 2017; Galafassi, 2018). However, this article is not discussing tactical activities, like recycling plastic, and renewable energy, or the creation of artwork, but rather how to make work itself more sustainable and how to help people think more sustainably. Helping people have a sustainable approach encourages sustainable tactical decisions and activities by the people who comprise our organisations more likely. Enabling a sustainable mindset is the step before the decision: what behaviours make someone more likely to make sustainable choices?

The link to sustainable behaviours is important for radical KM because radical KM seeks to make knowledge management take a strategic view of knowledge work from the perspective of the knowledge worker. Sustainability comes from making space for creativity in our organisations, so that they are not just analytical, rational, and process-oriented factories, but creative and innovative ecosystems.

## Sustainable Behaviours and Learning From Others

The value of knowledge management in the 21st century comes from learning, not from the databases and the documents of the 20th century, but from the experience of learning. It is necessary for knowledge management to take a more active role and to ask what are the desired sustainable behaviours and is there a group of people who consistently exhibit many of them that we can learn from? How do we focus less on databases and documents and more on continuous learning?

To address these questions, we need to understand the characteristics that make someone sustainable. Russel Reynolds Associates defined a sustainable leader as, “someone who looks beyond immediate, short-term gains to see the role their organization plays in a larger context. They set strategies and ensure the delivery of results that meet the triple bottom line of social, environmental, and financial performance” (Russel Reynolds Associates, 2016). The behaviours that they identified as sustainable include three categories: sustainable mindset, systems thinking, and relationship building. Sustainable mindset is made up of a sense of purpose; enlightened self-interest, considering others as well as ourselves; a long-term orientation; presenting and achieving highest potential while staying in the present moment; courage; integrity; open-mindedness; and transparency. While systems thinking is composed of seeing the bigger picture, appreciating the details, maintaining a balance, and keeping things simple. And, finally, relationship building is about understanding across cultures, appreciating and embracing diversity, network, meaningful dialogue, empowering stakeholders, and measuring improvements (Russel Reynolds Associates, 2016).

**FIGURE 2:**
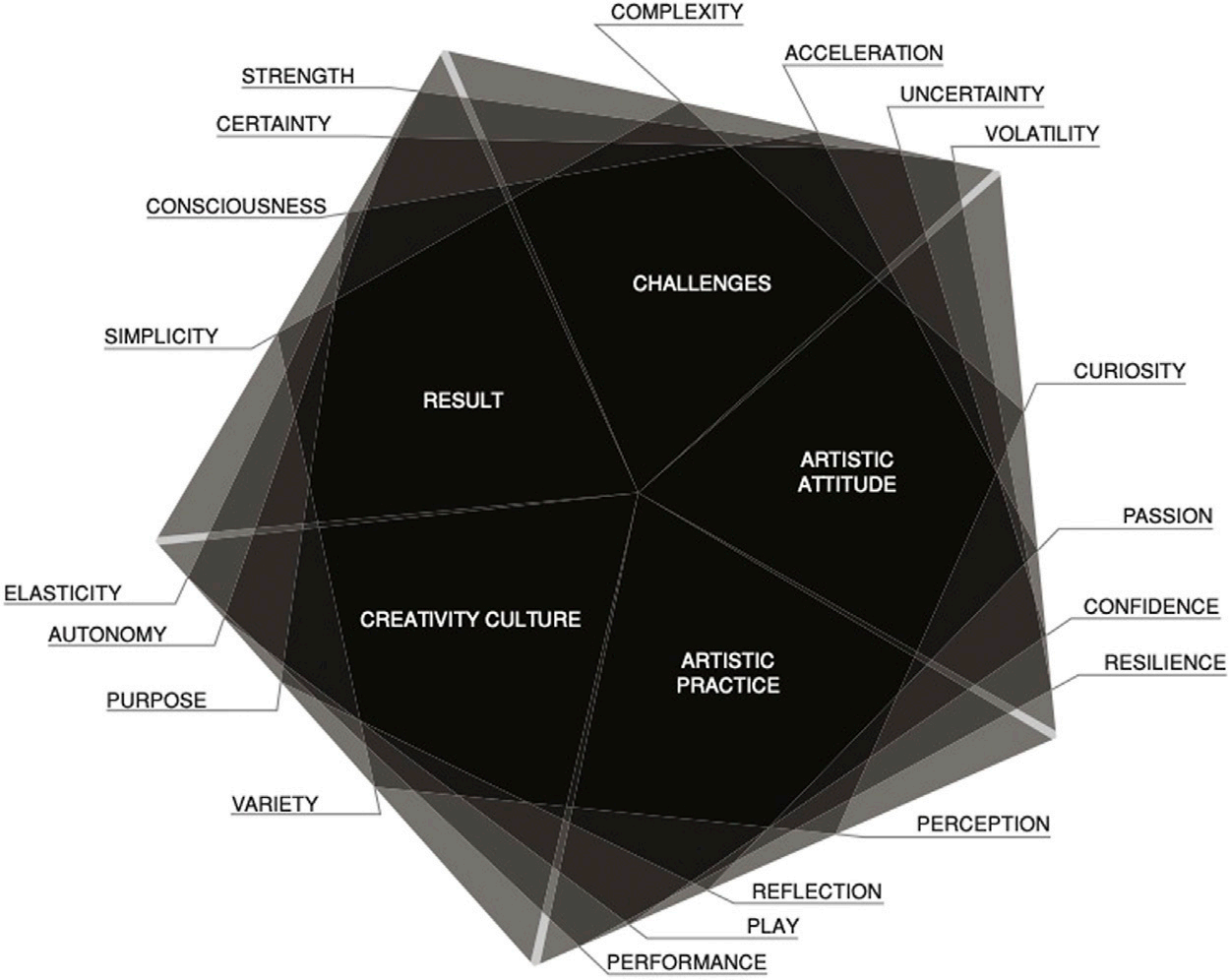
Age of artists, bloom (used with permission) (Dobiéy and Koeplin, 2020).

Is there a group of people who exhibit these behaviours? Many, if not all, of these behaviours are exhibited by artists and is researched and discussed extensively in the book *Creative Company: how artful creation helps organizations to surpass themselves* by Dirk Dobiéy and Thomas Koeplin. Dobiéy and Koeplin interviewed more than 100 artists and managers in performing their ethnographic research, which allowed them to create the model as in Figure 2.^3^

The model depicts the impact of an artistic attitude (being curious, passionate, confident, and resilient) combined with an artistic practice (perception, reflection, play, and performance) on the challenges that organisations face (complexity, accelerating change, uncertainty, and volatility). Applying artistic attitudes and practices to the challenges results in a culture of creativity where variety, purpose, autonomy, and elasticity are the norm. This creative culture in turn results in simplicity rather than complexity, consciousness rather than acceleration, certainty rather than uncertainty, and strength rather than volatility. In short, it creates an organisation that is agile and adaptable and able to cope with the constantly changing conditions with speed and grace because people are not restricted in their response but can use all their skills and abilities to accomplish the purpose of the organisation.

These learnings from artists, as depicted in the model, connect to the sustainability behaviours identified earlier in that they embody similar underlying mindsets: sustainability (the artist and their practice is, by necessity, sustainable; otherwise they and it would not exist), systems thinking (seeing the bigger picture while appreciating the details in creating their works), and the importance of relationships (understanding across cultures, diversity, dialogues, empowerment, and measuring improvements).

The answer to the final question “How do we focus less on databases and documents and more on continuous learning?” lies in adopting the playful, curiosity-infused behaviour of artists, which again ties into the desired sustainable behaviours and mindsets. It should first be noted, however, that knowledge management was never about databases and documents; those things and the technology that supports them are only enablers to knowledge management. Knowledge management has always been about learning and finding the knowledge that was needed to do the job. It just happens that now, because of the fluidity of our work environment, knowledge is less likely to be codified and more likely to be discovered by trial and error (Thomas and Seely Brown, 2011).

Having established the connection between sustainable and artistic behaviours, the next step is to consider the adoption of these behaviours in our organisations, through knowledge management and learning.

## Learning Like an Artist

The keys to learning like an artist are curiosity and iteration. These behaviours also arise through play, so we can also talk about learning through play and mean the same thing. However, there are additional personal development aspects that occur through art-based interventions, because art, for as many as 50% of the people, has bad memories associated with it (The Office, 2015). It is the observation of the author that in overcoming the fear of art, people are more confident in other areas of their lives; this finding is confirmed by research studies (Ippilito and Adler, 2016).

Art is used as a metaphor, to help people understand the ideas and processes around curiosity and iteration as well as the other desirable and sustainable behaviours identified previously. Art is also used to create depictions and representations of desired organisational behaviours and outcomes. The approach is transformative, but how does it happen and what happens in an art-based initiative (ABI)? That is described next.

## Using Art to Transform People and Organisations

ABIs are different from creative thinking, design thinking, or Agile (the software development methodology), although all of them use some aspect of creativity or an artistic approach in their implementation. Creative thinking is the ability to think about things in a new way, which incorporates perception, reflection, and curiosity from the artistic attitude and practice areas as in Figure 2 but does not use art in its implementation. The same is true for design thinking and Agile, although they both use the other aspects of artistic attitude and practice as well, but neither of them uses art per se, although both may use prototyping, which can come close to creating an artistic artefact. ABIs actually use artistic modalities, for example, drawing, painting, theatre, music, poetry, and writing/storytelling.

Darsø (2004), in her book *Artful creation: learning-tales of arts-in-business*, identifies four ways in which organisations use art.

1. Decoration;
2. Entertainment;
3. Instruments (for teambuilding, communication training, leadership development, problem-solving, and innovation processes); and
4. Strategic process of transformation (personal and leadership, culture and identity, creativity and innovation, as well as customer relations and marketing).

For the purposes of this article, the third and fourth uses are the focus, especially the fourth as we are discussing how to change the behaviour and help people and organisations become more sustainable and better able to cope with VUCA.

ABIs can take various forms: music, theatre, painting, drawing, sculpture, and improvisation are all available possibilities. The key to success is to not just do them once, but to have on-going support and to have the support come from people who understand business; these are not necessarily artists, and in fact people with both art and business skill sets are preferable (Darsø, 2016).

Berthoin Antal and Strauß (2016) identified 14 categories of added value that come from ABIs; they include everything from personal development and seeing things differently, through to the level-spanning effects of collaboration, experiencing the unexpected and new, and adapting ways of working that made people more able to deal with uncertainty and crisis. Importantly, they also noted that it made participants more aware of their emotions at work and they were better able to address them, thus enabling the premise of new work (Berthoin Antal and Strauß, 2016).

Giovanni Schiuma maps the connection between ABIs and value creation. He links ABIs to the knowledge assets they create through various processes for stakeholders, innovation, operations, and product processes, recognizing the development of strategic knowledge assets, enhancement of organisational capabilities, improvement of organisational performance, and ultimately delivering value to the stakeholders as a result. He documents this map so that management and practitioners have a clear relationship between ABIs and the value they create (Schiuma and Carlucci, 2016).

The literature cited supports the use of art for transformation and learning and recognises its importance in knowledge creation (one aspect of knowledge management). The following case studies further illustrates this connection.

## ABI Case Study: Danish Government

Meisiek and Barry present a case study of how ABIs can work in an organisational setting in their article, “Organizational Studios: enabling innovation”. In this situation, they studied the example of a Danish government organisation that was experiencing a lack of collaboration and knowledge sharing between the employees of two of its departments (law-making and citizen services). There were two main roadblocks to these two departments working more efficiently and effectively together: a silo mentality and internal rivalries. An innovation and knowledge sharing (IKS) department was established to address the situation. The IKS department initially tried some ABIs but the results, while positive, were short-lived.

In an effort to create long-lasting effects, the IKS department decided to create a studio with a team to support it. The team took a year to organise and setup the studio with an artistic inquiry process and artistic materials before running a pilot project. Much of the time and effort over the year it took to setup went into the creation of the artistic inquiry process so that it would support nonlinear thinking and help solve wicked problems by embracing complexity rather than by reducing it.

During the 5-day pilot, the participants used the available materials to create models and artefacts representing various principles and service ecologies that existed within the organisation. Through executing the activities over the course of the pilot, the participants became aware of behaviours that were detrimental to achieving not only the goal of the assignments, but also the objectives of their work and sharing and collaborating together. To overcome this situation, facilitators encouraged “what if” questions and challenged assumptions and the ways people were working. The conversations that arose from these questions triggered reflection and sense-making processes that took the participants towards new and different directions.

In the end, the results of the pilot were positive and enabled the participants to develop a new and innovative solution to the problem they were addressing in the pilot workshop. The studio remained in place for the IKS team to use with other teams who needed to develop new products and solve problems using creativity and abstract thinking. The ABIs were not a one-off experience but an on-going tool that allowed teams to come together, transforming the way they worked together as well as the solutions they developed (Meisiek and Barry, 2016).

## ABI Case Study: Xerox PARC Artist-in-Residence Program

During the 1990s, Xerox PARC implemented an Artist-in-Residence programme that connected working artists with research scientists. The programme was planned to run as a trial for one year, but it was so successful that it ran for six years. The artists asked questions and wanted to do things with the technology that the researchers were developing that the researchers had never thought about. In turn, the artists created artwork that would not have been developed without the technology. In working together, the artists and the scientists enabled their own creativity and willingness to experiment (VanGundy and Naiman, 2005).

## ABI Case Study: Equiva Services

Equiva Services (a support services company in the oil and gas sector) used ABIs to learn and create new knowledge. Participants went on field trips to gather information about “new economy” companies and then returned and built sculptures to give form to their ideas about how to bring “new economy” thinking into their “old economy” organisation. The process created curiosity, dialogue, storytelling, and reflection into the process and allowed them to realise their ideas in a simple, yet powerful way (VanGundy and Naiman, 2005).

## ABI Case Study: LexisNexis

LexisNexis (a company that provides computer-assisted legal research as well as business research and risk-management services) uses improv in their corporate training programme. Using improv helps teach skills traditionally thought of as “soft skills”, things like communication and empathy. Learning these skills impacts people’s ability to work and collaborate together to deliver projects successfully. The organisation has improved team effectiveness through this use of improv (VanGundy and Naiman, 2005).

## ABI Case Study: Conclusion

The organisations in each of these case studies have used creative methods, whether it be improv/theatre, 3-D art, or fine arts, to create work environments that include more of the sustainable behaviours identified earlier in this article, for example, presencing, courage, open-mindedness, systems thinking, and relationship building. While they may have set out to improve team-building or innovation, the behaviour changes from the ABI and adopting an artistic attitude and practice has wider impact.

## Conclusion

This study provides the basis for a next step towards embracing radical KM. It has made novel connections between creativity, sustainability, and new work, as the missing piece of the organisational puzzle. The author recommends further research. First, identify organisations that are interested in transforming their organisational culture to include a more balanced, sustainable approach, and then initiating various forms of ABIs, collecting data, and anecdotes as the research progresses. This ultimately leads to case studies and further publications supporting or disproving the ideas discussed in this article.

Our organisations have focused on the division of labour, the compartmentalisation of knowledge, and treating knowledge work like it is part of a production line. Anchoring our organisations in these industrial-age paradigms has left out space for creativity and does not suit desired knowledge-age requirements. People are not machines, knowledge work requires different behaviours to support it and be successful, and it requires the behaviours that have been ignored in favour of efficiency and effectiveness. Making space for creativity through ABIs reactivates these behaviours and enables a sustainable approach to work; examples like the case study from the Danish government support this approach for knowledge management.

Echoing Ippolito and Adler’s summation from their article, “*From Aspiration to Evidence*” (Ippolito and Adler, 2016), it is time to do things differently, embracing the things that have been forgotten, ignored, and laid aside. Encouraging people to use all of their creative and analytical skills by incorporating art and artistic practice back into our organisations is a way of moving forward in a sustainable, holistic way. As quoted by Einstein, “we cannot solve problems by using the same kind of thinking we used when we created them”. It is time to embrace uncertainty and chaos through the use of artistic attitudes and practices and move boldly and sustainably into the 21st century. It is time to embrace radical KM.

## Data Availability

The original contributions presented in the study are included in the article/supplementary material; further inquiries can be directed to the corresponding author.

## Footnotes

1 The literature review was based on the article, “Literature review as a research methodology: An overview and guidelines” (Snyder, 2019), and the approach used for this article is categorised as *Examples of Contribution* as described in Snyder’s article. As such, the literature review executed by the author included reviewing the list of references from the book *Creative Company* (Dobiéy and Koeplin, 2020) and numerous Google searches looking for academic and other reputable publications using combinations of the search terms ABI, arts-based initiative, arts-based interventions, creativity, innovation, knowledge management, sustainability, sustainable leadership, sustainable behaviours, new work, new work behaviours, and artist-in-residence.

2 New work is defined as the new way of working in the global and digital age. The term was originally coined by Frithjof (Bergmann, 2004) in his book *Neue Arbeit, Neue Kultur*, and embodies the core values of autonomy, freedom, and participation in the community.

3 The author did not contribute to the creation of the model; it is useful in describing what can be learned from artists and the impact on the organisation. To understand how the model works, read it by going clockwise starting at challenges, which can be addressed using artistic attitude and artistic practices to create a creative culture and producing results. Each section of the pentagon is composed of four elements, for example, challenges is composed of complexity, acceleration, uncertainty, and volatility that correspond with the elements in the other sections. For example, complexity is addressed through curiosity and perception, leading to variety, and ultimately resulting in simplicity. It is not the purpose of this article to explain or argue for or against the model developed by Dobiéy and Koeplin. The purpose for including the model is to illustrate what can be learned from artists and applied organisationally.
